# Multifunctional Cosmetic Containing Blueberry and Tinosorb M®-Loaded Microparticles Improves Sunscreen Performance

**DOI:** 10.15171/apb.2019.027

**Published:** 2019-06-01

**Authors:** Daiana Schiavon, Daniela Novello Martini, Gabriela Brocco, Júlia Scherer Santos, Ana Paula Anzolin, Luciana Grazziotin Rossato-Grando, Hamid Omidian, Charise Dallazem Bertol

**Affiliations:** ^1^Curso de Farmácia, Universidade de Passo Fundo, Passo Fundo, Brasil.; ^2^Curso de Farmácia, Centro Universitário União Dinâmica das Cataratas, Foz do Iguaçu, Paraná, Brasil.; ^3^Programa de Pós-Graduação em Envelhecimento Humano, Universidade de Passo Fundo, Passo Fundo, Brasil.; ^4^Programa de Pós-Graduação em Bioexperimentação, Universidade de Passo Fundo, Passo Fundo, Brasil.; ^5^Pharmaceutical Sciences, Nova Southeastern University, Fort Lauderdale, Florida, USA.

**Keywords:** Sunscreen, Blueberry, Microparticles, Stability, Tape stripping

## Abstract

***Purpose:*** We aimed to evaluate the effect of blueberry extract and microparticles (MP) on sunscreen performance of multifunctional cosmetics. Octocrylene (OCT), benzophenone-3 (BENZ-3) and Tinosorb® M (MBBT) were employed as UV filters.

***Methods:*** An *in-silico* modeling was used to determine the UV filters concentrations to obtain high values of sunscreen protection factor (SPF) and UVA protection factor (UVA-PF). MBBT and blueberry-loaded microparticles (MP_MB+B_) and MBBT-loaded microparticles (MP_MBBT_) were prepared by spray-drying. OCT and BENZ-3 were added in the oil phase of cosmetics. Cosmetics A and B contained MP_MB+B_ and MP_MBBT_, respectively, and cosmetic C was prepared without MP. Characterization, physicochemical stability and *in vitro* SPF was performed. UV filters distribution in human stratum corneum (SC) for each cosmetic was performed. Anti-oxidant activity of blueberry extract was evaluated.

***Results:*** Sunscreen combination with the highest SPF was selected for formulations. Formulations A and B maintained their rheological behavior over time, unlike formulation C. *In-vitro* SPFs for formulations A, B and C were 51.0, 33.7 and 49.6, respectively. We also developed and validated a method for analysis of the UV filters by HPLC/ PDA suitable for the *in-vivo* assay. In Tape stripping test, MBBT showed SC distribution similar for all cosmetic formulations. OCT and BENZ-3 distribution to formulation A and C was also similar. Blueberry extract showed antioxidant capacity of 16.71 μg/mL equivalent to vitamin C.

***Conclusion:*** Cosmetics containing MPs presented better physical stability. Blueberry increased the photoprotective capacity of the formulations and added extra benefits due to its anti-oxidant and anti-aging properties.

## Introduction

A good sunscreen is expected to block the UV penetration and to prevent its acute and chronic damages and ultimately skin cancer.^1^ Sunscreens are characterized by their sunscreen protection factor (SPF) and physiochemical properties. Apart from the active ingredient, a sunscreen may contain other additives such as moisturizing agents, antioxidants, and repellents which enhance its photoprotective properties and encourage its frequent use.^1^ Sunscreens containing natural anti-oxidants are in line with the current trend of developing multifunctional cosmetics or “smart” products that can offer extra benefits such as antioxidant and anti-inflammatory properties.^2-5^

Blueberry (*Vaccinium* sp.) from Ericaceae, is known as the fruit of longevity due to its high polyphenols and anthocyanins (mainly delphinidin and malvidin) content with antioxidant function^6^ as well as vitamins, minerals, and resveratrol among others.^7^ Besides its anti-aging function, polyphenol can increase the photoprotective potential of the formulations.^8^ Blueberry is a potential anti-cancer^9-11^ and can reduce tumor proliferation in murine melanoma cells^12^ and reduce photoaging effect and free radical generation caused by UVB radiation on human dermis cells^13^ and on keratinocytes.^14^

In sunscreen formulations, MPs can delay the penetration of the active, allowing photoprotection or a longer period of action.^15,16^ Moreover, they increase photostability, protect against oxidation, reduce odors of compounds, avoid incompatibilities, and reduce allergies and dermatitis caused by sunscreens.^16,17^

This research intended to develop and evaluate the effect of blueberry and the role of MPs in three multifunctional cosmetics sunscreen formulations performance. Sunscreen formulations was composed of Octocrylene (OCT), Benzophenone-3 (BENZ-3), and Tinosorb^®^M (MBBT - Bisoctrizole or 2,2’-methylene-bis-6-(2H-benzotriazol-2-yl)-4-(tetramethyl-butyl)-1,1,3,3-phenol).

## Material and Methods

### Determination of the concentration of the UV filters and SPF / UVA-PF in-silico

The amounts of OCT, BENZ-3 and MBBT used in the formulations were at the permitted levels.^18^ Different concentrations of three UV filters were evaluated by the *in-silico* Online BASF Sunscreen simulator, obtaining solar protection factor for UVB (SPF) and protection factor for UVA (UVA-PF). Combination 1 was made of 3% OCT, 6% BENZ-3 and 6.4% MBBT (total of 15.4% UV filters), and Combination 2 was made of 6% OCT, 8% BENZ-3 and 10% MBBT (total of 24% UV filters).

### Materials

The compounds used in the formulations were purchased in different suppliers. OCT (98.4% v/v, supplied by CosmeTrade Commercial, Porto Alegre/RS/Brazil), MBBT (59.42% w/v, D’Altomare Química, Santo Amaro/SP/Brazil, Manufacturer BASF), BENZ-3 (99.80%, Audaz São Paulo /SP/Brazil), blueberry extract (9.68% of anthocyanins, Viafarma Supplier, Manufactured by Quimer, São Paulo/SP/Brazil), hydroxypropyl methylcellulose (HPMC) (Methocel K15M^®^, Colorcon, Cotia/ SP), butylhydroxytoluene (BHT) (Alpha Química, Porto Alegre/RS), ethylenediamine tetra acetic acid (EDTA) (Synth, Diadema/SP), imidazodinylurea (Audaz Brasil, São Paulo/SP), octyl stearate (Alpha Química, Porto Alegre/RS), polysorbate 80 (Neon) and Lanette N (Alpha Química, Porto Alegre/RS) were used. Solvents and reagents used include N, N-dimethylformamide (Dynamic), methyl alcohol (Dynamic), acetonitrile high-performance liquid chromatography (HPLC) grade (Sigma-Aldrich), glacial acetic acid (Audaz, Brasil, São Paulo/SP), and ultra-pure water in a Direct-Q^®^ system (Millipore, USA).

### Preparation of microparticles (MPs)

The concentrations of the components were defined considering the maintenance of the same concentration of UV filters in all formulations providing an adequate SPF/UVA-PF (see in the silico mathematical modeling). The other compounds followed the permissible concentrations in the legislation, and are commonly used. Pilot formulations were developed to obtain a homogeneous formulation capable to incorporate the compounds.

Two MPs formulations named MP_MBBT+B_ and MP_MBBT_ (without blueberry) were prepared and composed of HPMC, polysorbate 80, MBBT and blueberry (B) and water to make a 100% composition (according to Table 1). Initially, three distinct phases were prepared in water-bath under heating at 70 ° C and stirring for 90 min and then pooled together.

**Table 1:** Composition of blueberry-loaded microparticles (MP_MBBT+B_) and MBBT-loaded microparticles (MP_MBBT_)

	**Components**	**MP**_MBBT+B_	**MP**_MBBT_
Phase 1	HPMC Water	0.25% up to 30%	0.25% up to 30%
Phase 2	MBBT Polysorbate 80 Water	6% 3% Up to 30%	6% 3% up to 30%
Phase 3	Blueberry Extract Polysorbate 80 Water	2.5% 3% Up to 40%	- 3% up to 40%

A spray dryer (LabMaq, MSD 1.0) was used to dry the samples and to obtain MPs (inlet temperature of 115ºC, flow rate of 0.6 L/h). The vials containing samples were sealed and stored in a desiccator followed by drying. A scanning electron microscopy (SEM) (Vega LM3/Tescan Oxford EDS Instrument) was used to study morphology. Samples were mounted with carbon adhesive on an aluminum holder, covered with gold in a metallizer Quorum (Q150R ES), and photographed at 20 kV.

### Preparation of multifunctional cosmetic sunscreens containing UV filters and blueberry extract

Three emulsions formulations were prepared and named as formulation A, B and C. Formulations A and B presented MPs in their composition. Formulation C did not contain any MPs. UV filters and the additives content were kept constant in all formulations. Blueberry extract was present in the formulations A and C (Table 2).

**Table 2:** Composition of multifunctional cosmetics A, B, and C

	**Components**	**A**	**B**	**C**
Phase 1	Ethylenediamine tetra acetic acid (EDTA), %	0.11	0.11	0.11
	Imidazodinylurea 50% (w/v), %	0.6	0.6	**-**
	MBBT, %	**-**	**-**	20
	Polysorbate 80, %	**-**	**-**	3
	Water, %	45.50	48.00	23.17
Phase 2	Octyl stearate, %	3	3	3
	Lanette N, %	8	8	8
	OCT, %	6	6	6
	BENZ- 3, %	8	8	8
	Butylhydroxytoluene (BHT), %	0.05	0.05	0.05
Phase 3	MP_MBBT+B_ %	28.75^a^	**-**	**-**
	MP_MBBT_ %	**-**	26.25^b^	-
	Blueberry extract, %	**-**	**-**	2.5
	Polysorbate 80, %	**-**	**-**	3
	Water, %	**-**	**-**	23.17

^a^2.5g blueberry extract, 20 g MBBT, 6 g polysorbate 80, and 0.25 g HPMC. ^b^20g MBBT, 6 g polysorbate 80, and 0.25 g HPMC.

The components were weighed, separated according to the phase, and heated to 70-75°C. The aqueous phase 1 was composed of EDTA, imidazodinylurea, MBBT, polysorbate 80 and water. EDTA was used as chelating agent, imidazodinylurea was employed as the preservative, MBBT as UVA/UVB filter, polysorbate 80 was employed as surfactant in order to increase MBBT solubilization. The phase 2 contained octyl stearate, OCT, BENZ-3, BHT and water. Octyl stearate was used as solubilizer of OCT and BENZ-3. OCT and BENZ-3 were used as UVB filters. BHT was applied as antioxidant to prevent blueberry oxidation. Phase 1 was poured into the phase 2 and vigorously stirred. Phase 3 was added to formulations A, B and C previously prepared. MP powder MP_MBBT+ B_ and MP_MBBT_ were added to formulation A and B respectively. Regarding to emulsion C, phase 3 was prepared under heating and stirring and then added to formulation C.

### Characterization and physicochemical preliminary stability assessment of multifunctional formulations A, B and C

The formulations A, B and C were characterized for their organoleptic characteristics, pH at 5% (w/v, in water) (potentiometer Digimed), centrifugation test (1g of each formulation subjected to 3000 rpm for 30 minutes in Centribio centrifuge), and rheological behavior. Samples were evaluated in triplicate at time 0 (immediately after preparation) and 90 days post-preparation.^19^ During the study, the samples were stored at 25 ± 2°C, relative humidity of about 60% ± 2°C, protected from light.

To study their rheological behavior, a rotational viscometer (Brookfield RVDV II) was used over the shear rates of 0-100 rpm, and the rheograms were further evaluated using a mathematical modeling.

Normality was assessed by D’agostino Pearson’s test. The results were analyzed by one-way ANOVA (for pH test) and two-way ANOVA (for the viscosity test) followed by the Tukey test (*P* ≤ 0.05).

### Determination of in-vitro SPF of cosmetics

Formulations (0.2 mg/mL) were prepared in 92% ethanol (v/v in water). The absorbances were read in a spectrophotometer (Cirrus 80 ST, FEMTO) every 5 nm, starting at 290 and extending to 320 nm. The *in-vitro* SPF was then calculated according to the following equation^20^:

$$SPF=FC.\sum_{290}^{320}E(\lambda ).ABS(\lambda )$$

Where FC is the correction factor, E (λ) is the erythematogenic effect of wavelength radiation (λ), Ι (λ) is the intensity of sunlight at (λ); and ABS (λ) is the spectrophotometric reading of the absorbance of the solution formulation (λ).

FC used was equals 10 to allow the obtainment of SPF of 4 by spectrophotometry for a cream containing 8% homosolate. The values of E multiplied by I (E X I) were previously set^20^ at each 5 nm, from 290 to 320 nm. For each λ analyzed, the absorbance is multiplied by the E X I value and, finally, multiplied the obtained value by the FC. Once this calculation has been made for each λ, the sum of all values is taken.

Normality was assessed by D’agostino Pearson’s test. Results were analyzed by one-way ANOVA followed by the Tukey test (*P* ≤ 0.05).

### Chromatographic conditions and method validation

Chromatographic conditions were optimized to provide a simple and reliable method capable of analyzing the UV filters used in the sunscreen formulations. A HPLC (Flexar LC Perkin Elmer, Burnsville, MN, USA) equipped with a binary pump, a PDA detector (fixed at 325 nm), and an autosampler (injection volume of 20 μL) was used. Peak areas were integrated into Chromera Workstation software. A C8 analytical column (NST nano separation technologies) (250 mm × 4.6 mm × 5.0 μm) was used. The mobile phase (acetonitrile (A) and water mixed with acetic acid (B) (pH 3.5)) eluted in a gradient mode as follows: 90:10 (A:B) ratio from 0-7 minutes, 100% A from 7-20 minutes, and 90:10 (A:B) ratio from 20-23 minutes. The flow rate was 1 mL/min, and the total run was 23 minutes.

For the validation, we considered specificity, linearity, limits of quantification (LQ) and detection (LD), precision and accuracy.^21,22^

### In-vivo UV filters quantification from multifunctional formulations in stratum corneum (SC) by tape stripping

*In-vivo* assays were performed to determine cutaneous penetration of the sunscreen formulations A, B and C. Ten male/female volunteers 18-50 years old with skin phototype II, III, IV were included in this study. The exclusion criteria were volunteers allergic to sunscreens/the components of the formulations/ adhesive tape, those using any skin sensitizing medication, and those with dermatoses, skin cuts in the area of application, and previous history of skin cancer. Based on previous studies,^23,24^ the contact time of the formulations with the skin was set at 30 minutes. Volunteers washed their forearms with water and neutral soap, and an area of 4 cm² was prepared for the application. All volunteers received a dose of 2mg/cm^2^ of each formulation at different sites of their forearm.

After 30 minutes, the Tape Stripping technique was performed to remove the SC. With a little pressure, a piece of tape was placed on the area containing formulation and it was later removed. This cycle was repeated using 5 pieces of adhesive tape.^25^ The tapes were placed in a beaker containing an approximately 10 mL of N, N-dimethylformamide diluent, and sonicated for 10 minutes. The volume was adjusted using a 10 mL volumetric flask, filtered and transferred to HPLC. After the process, the volunteers washed the forearm to remove the products.

Normality was assessed by D’agostino Pearson’s test. The results were analyzed by one-way ANOVA followed by the Friedman test (*P* ≤ 0.05).

### Antioxidant activity of the blueberry extract

The sequestering activity of DPPH (1,1-diphenyl-2-picrylhydrazyl) radical was determined.^26,27^ DPPH (0.1 mM) was prepared in 80% methanol (v/v in water). A 0.1 mL aliquot of the blueberry extract (1 mg/mL in 80% methanol) was added to 2.9 mL of the DPPH, homogenized and kept at room temperature in the dark for 30 min. Using a UV spectrophotometer (Lambda 20/ Perkin Elmer spectrophotometer), the absorbance was measured at 517 nm. A solution containing methanol and DPPH was used as control. The calibration curve was obtained with vitamin C solutions (40.0-120.0 μg/mL). The sequestering activity of the DPPH radical by the extract was expressed as μg/mL antioxidant capacity equivalent to vitamin C.

## Results and Discussion

### In-silico determination of the concentration of the UV filters and SPF/UVA-PF

*In-silico* and *in-vitro* tests are important tools in the development of sunscreens to determine preliminary qualitative and quantitative concentrations of the UV filters.^28^ The use of BASF Sunscreen Simulator showed good correlations with *in-vivo* results, representing a valuable tool in the development of sunscreens.^29^

Radiation blocking capability for the combinations 1 and 2 were 96% and 98%, respectively. The *in-vitro* UVA-PF and *in-vivo* UVA-PF for the combinations 1 and 2 were 13/16 and 23/21, respectively. Critical wavelength for both combinations was 380 nm, a wavelength showing suitable UVA protection. Combination 1 and 2 presented SPF of 30 and 50, respectively. The combinations 2 presented a higher SPF/UVA-PF due to the higher UV filters concentrations. For this reason, this combination was selected for further studies. The legislation requires at least an SPF of 6, an UVA-PF corresponding to 1/3 of the SPF and a critical wavelength of at least 370 nm^5^ which were found in both combinations. The UV filters selected for this study are safe; OCT is a liposoluble and photostable; BENZ-3 is a commonly used UVB filter; and MBBT is a photostable sunscreen with low risk of skin penetration.^28,30^

### Characterization of MBBT and blueberry-loaded microparticles (MP_MBBT+B_) and MBBT-loaded microparticles (MP_MBBT_)

Initially, in an attempt to prepare MP containing the three UV filters used in this study, we employed the solvent evaporation methodology. We prepared an emulsion. In the oily phase, we added OCT and BENZ-3. In the aqueous phase we added MBBT and blueberry extract. However, during the use of rotary evaporator to remove acetone used as solvent, a sticky composition was obtained.

In the technique used in this work, only the MBBT and the blueberry extract were microencapsulated. Before drying, the MP were milky in appearance and runny. MP_MBBT+B_ and MP_MBBT_ were slightly pink and white, respectively. The MP turned into a cohesive mass due to the presence of MBBT.

**Figure 1:**
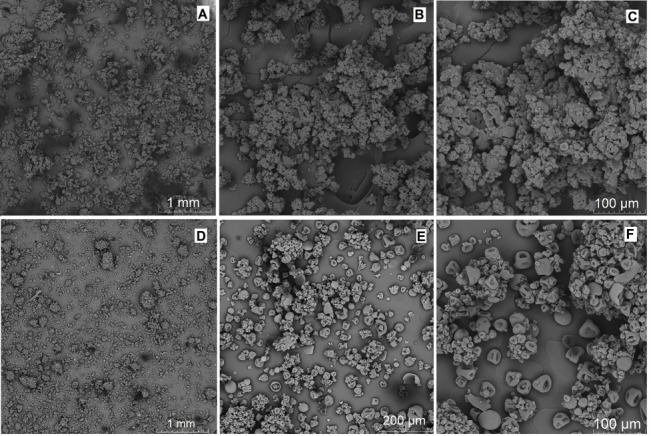
SEM of the microparticles containing 2.5% of blueberry extracts and 20% MBBT (A, B, C) (MP_MBBT_+B), and white microparticles containing 20% MBBT (D, E, F) (MP_MBBT_) at 100× (A, D), 500× (B, E) and 1000× (C, F) magnifications.

Figure 1 shows the SEM micrograms of both compositions. Formulation MP_MBBT+B_ showed microspheres characteristics with the roughest and most clumped wall, while MP_MBBT_ (without blueberry extract) had smoother surface with characteristics similar to microcapsules. The size of MP_MBBT+B_ was in the range of 100 to 200 µm, because these MPs formed cluster. The size of MP_MBBT_ was lower than 100 µm (Figure 1).

### Characterization, and physiochemical preliminary stability assessment of multifunctional sunscreen formulations

Formulations were homogenous and those containing blueberry were slightly pink. All formulations were easy to spread on the skin and had a characteristic odor of sunscreens. All pH values were in the range from 7.00 to 7.60, compatible with the area of application. Statistically, there was no pH difference between formulations at time 0 and after 90 days. The centrifugation behavior of the samples was also the same after 90 days as evidenced by no phase separation.

**Figure 2:**
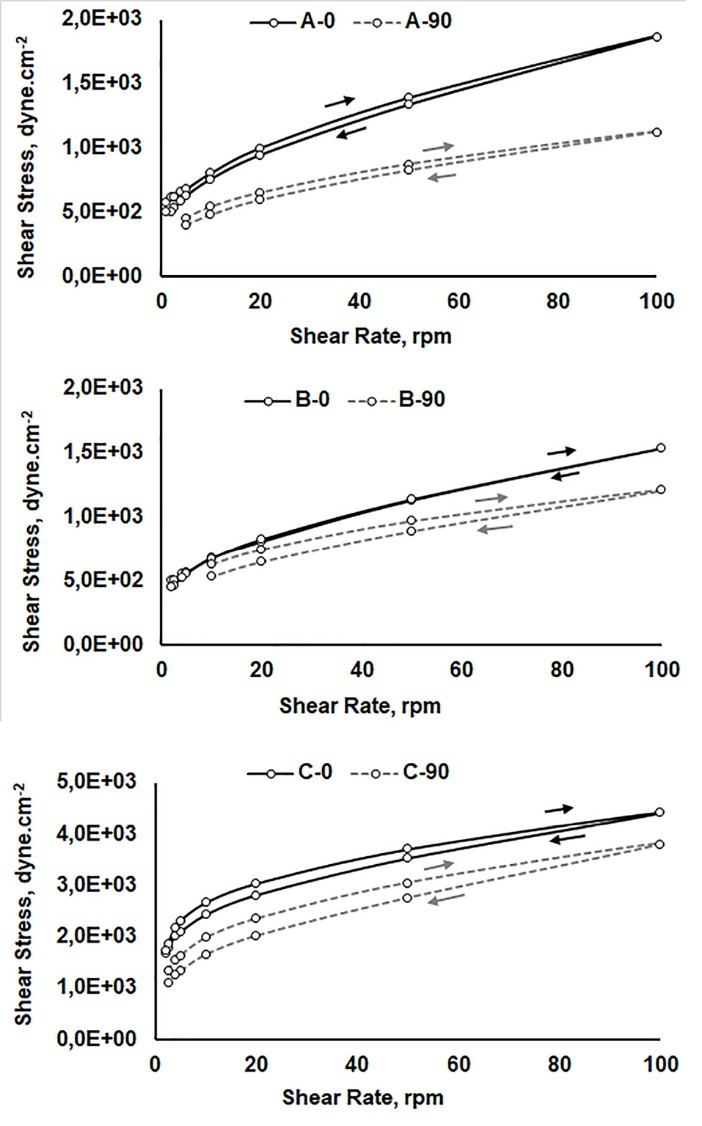
Rheograms of the sunscreens A (top), B (middle) and C (bottom) show hysteresis (thixotropic behavior) immediately after preparation o days and 90 days post-preparation, except for B immediately after preparation.

Figure 2 shows general rheological behavior of the multifunctional sunscreens formulations at the shear rates of 0-100 rpm, at the time of preparation and ninety days post-preparation. Formulations A and B remained stable after ninety days, and the formulation B showed superior stability compared to the formulations A and C. The rheogram also shows that the yield value of the formulations C was three times higher than those of the two other formulations, suggesting that the formulations C had a greater spreadability. However, the formulation C statistically experienced decrease in viscosity after 90 days, demonstrating the decrease of physical stability during the study. Formulations A and B displayed a very similar flow behavior. All three formulations showed a yield value (plastic flow) followed by a pseudoplastic flow which was more notable in the case of formulations C. All three formulations displayed hysteresis over the range of the shear rates studied, and the hysteresis was greater for the formulations C compared with the other two. Formulation B didn’t present hysteresis immediately after preparation. All three formulations showed thixotropic behavior, a desirable feature promoting greater photoprotection.^31^

As showed in Table 3, rheological behavior of the Formulations A and B best fitted a Casson model while formulation C best fitted as Ostwald model. Formulations containing MPs presented plastic behavior and the formulation without MPs presented pseudoplastic behavior. The presence of MPs therefore, altered the rheological behavior. Nevertheless, both models have been previously used for sunscreen formulations.^31,32^ Pseudoplastic behavior promotes the formation of a homogeneous film on the skin, ensuring adequate sun protection.^31^

**Table 3:** Mathematical modeling of the rheological behavior of cosmetics sunscreens

**Models**		**Freshly prepared**			**After 90 days**		
	**Equations**	**A**	**B**	**C**	**A**	**B**	**C**
Bingham	*τ =τ*_0 +_ *η.γ*	0.9740± 0.0065	0.9764 ± 0.0063	0.9335± 0.0138	0.9704 ± 0.0055	0.9764 ± 0.0057	0.9337 ± 0.0232
Casson	*τ* ^ 0.5 ^ *= τ*_0_^ 0.5 ^ *+ η* ^ 0.5 ^ *.γ* ^ 0.5 ^	0.9969 ± 0.0009	0.9978± 0.0006	0.9804 ± 0.0074	0.9956 ± 0.0018	0.9966± 0.0020	0.9792 ± 0.0107
Ostwald	*τ=K.γ* ^n^	0.9439 ± 0.0260	0.9706 ± 0.0198	0.9948 ± 0.0044	0.9925 ± 0.0022	0.9965 ± 0.0020	0.9984 ± 0.0008
Herschel–Bulkley	*τ= τ*_0_ *+ K.γ* ^n^	0.7665± 0.0683	0.8487 ± 0.0113	0.7741 ± 0.0133	0.8992 ± 0.0363	0.9907 ± 0.0088	0.8446 ± 0.0787

Where *τ* is the shear stress; *τ*_0_ is the critical shear stress; *η* is the viscosity; *γ* is the shear rate, *K* is the consistency and *n* is the power law index. Results are expressed as regression coefficient (n = 3, mean ± SD).

### Determination of the in-vitro SPF of formulations

The *in-vitro* SPFs for the formulations A, B and C were 41.51±3.48, 33.65±2.67, and 49.58±2.83, respectively. All formulations were statistically different. The blueberry extract present in the formulations A and C improved the SPF. MPs did not improve the SPF. The *in-vitro* SPF technique does not allow detecting the interaction of the particles with the skin, including the formation of a film that acts as a physical filter.^33,34^ In this way, this assay should be used with other tests to demonstrate the effectiveness of the MP sunscreens.

### In-vivo UV filters quantification from multifunctional formulations in stratum corneum (SC) by tape stripping

The effectiveness of the sunscreens is determined by the SPF and UVA-PF.^28^ The official test to determine the SPF is to expose the volunteers to UV radiation. By exposing an unprotected area of the skin to a UV light, and covering another area with sunscreen, the erythematous dose (for UVB radiation) or the minimum pigment dose (for UVA radiation) can be determined.^5,28^

The tape stripping technique can also be used for *in-vivo* evaluation. This technique can quantify the amounts of the active substance retained in the SC and is minimally invasive because the removed SC can rapidly be reconstituted without damaging the epidermis and dermis.^35^ The SC corresponds to the target site of sunscreens. Therefore, tape stripping can be used to predict sunscreen efficacy.

Statistically the MBBT results showed a normal distribution while OCT and BENZ-3 were not normal. The amounts of the MBBT found in the SC was similar among the formulations A, B and C.

**Figure 3:**
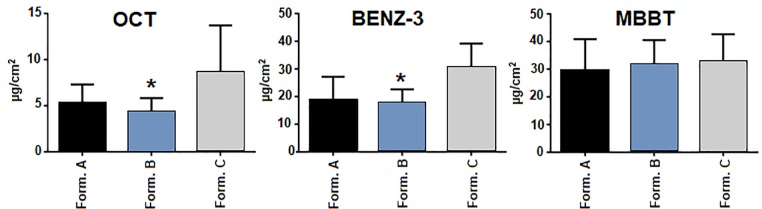
*In-vivo*quantification of the UV filters in sunscreen formulations A, B and C on human skin by tape stripping. * Formulation B and C were different. (MBBT: normal distribution by D’agostino Pearson, one-way ANOVA showed no differences; OCT and BENZ-3: non-normal distribution by D’agostino Pearson, B and C are different by the Friedman test, *P* < 0.05).

Therefore, the presence of MPs did not influence MBBT SC distribution. Regarding to BENZ-3 and OCT SC distribution from formulation A and C a similar profile was observed, which is expected since BENZ-3 and OCT are not microencapsulated. Unlike, BENZ-3 and OCT SC distribution from formulation B was statistically lower. The presence of blueberry may have affected OCT and BENZ-3 content in SC since those UV filters showed a similar distribution in formulations A and C (Figure 3).

Cosmetic formulations containing MP_MBBT+B_ showed a similar skin profile distribution to cosmetic containing non-microencapsulated ingredients. Despite this, topical application of MP formulations may increase protection against erythema^17^ fulfilling the role of MP systems as suitable for sunscreens.

### Antioxidant activity of blueberry extract

Using a DPPH assay, it was found that blueberry extract has an antioxidant capacity with a value of 16.71 μg/mL equivalent to vitamin C, indicating a potential anti-aging action. In the *in-vitro* SPF assay, it was observed that the blueberry extract also improved the photoprotective capacity of the formulations.

## Conclusion

Multifunctional cosmetics sunscreen were successfully prepared. All formulations displayed pH skin compatible, a combination of plastic flow, pseudoplasticity and thixotropy, a very desirable flow property in the preparation, application, and performance of sunscreen formulations. Cosmetics containing blueberry and MBBT-loaded MPs showed anti-oxidant activity and improved physicochemical stability. All formulations presented high values of SPF, mainly, the cosmetic containing blueberry and MBBT-loaded MPs. MPs increased the stability and the blueberry increased the photoprotective and anti-oxidant capacity. Therefore, cosmetic containing MBBT and blueberry-loaded MPs presented the best performance as sunscreen.

## Ethical issues

The work was approved by the Research Ethics Committee according to approval number: 58224116.0.0000.5342, and the volunteers signed the consent form. Procedures were in accordance with the ethical standards of the responsible committee on human experimentation (institutional and national) and with the Helsinki Declaration.

## Conflict of Interest

The authors report no conflict of interest.

## Acknowledgements

This work was supported by Project “Implantação do Laboratório de Desenvolvimento e Avaliação de Alimentos Funcionais e Nutracêuticos – NUTRA_ALI” funded by Programa Gaúcho de Parques Científicos e Tecnológicos – Programa PGtec.
